# Ageing and ethical challenges in physiotherapy: application of the RIPS model in ethical decision-making

**DOI:** 10.1080/07853890.2021.1896437

**Published:** 2021-09-28

**Authors:** José Luís Sousa, Sónia Gonçalves-Lopes, Verónica Abreu

**Affiliations:** a Escola Superior de Saúde Jean Piaget de Vila Nova de Gaia; b Research in Education and Community Intervention (RECI), Instituto Piaget

## Abstract

**Introduction:**

The population aging and consequent epidemiological changes in elderly population create new challenges for physiotherapists’ intervention and their decision-making process [1]. Ethical issues are one of these challenges [2]. It is required from the physiotherapist special attention to the fragility that can represent the process of ageing in the lifespan of the elderly [3,4] and possible threat of paternalistic attitudes form health care professionals or family and their likely consequence, the reduction autonomy [5,6]. Difficulties in decision-making on ethical issues may be due to a possible conflict between respect for professional ethics values and care systems values including the family [3,4,6]. The Realm-Individual Process-Situation (RIPS) model is an ethical decision-making addressed to physiotherapist [7]. The decision-making process has 4 steps: recognising and defining the ethical issues, reflect, decide the right thing to do, and implement, evaluate, re-assess. So, the aim of this work is to apply the RIPS model to a clinical case in order to demonstrate the applicability of this ethical decision-making model involving the elderly.

**Materials and methods:**

Case study of a 75 years old man that comes from a Central Hospital to a long-term facility. He is alert and oriented. During the admission process, the Physiotherapist (PT) asked the patient to describe the motive for their initial hospitalisation. The patient's process had not yet arrived at the facility. The patient reported that he had developed shortness and weakness and admitted for evaluation. He said that in the hospital they said that he was weak and needs physiotherapy before going home. The family asked to speak with the PT privately and reports that he has been diagnosed with a stage 4 inoperable lung cancer with few weeks of life. The family didn’t share this information with the patient to not upset him and they don’t want any one of the staff to tell him about their diagnosis. The PT bring this issue to the attention of the “ethical board”. Written informed consent was obtained.

**Results:**

The analysis of the facts that surround this case, in the step on PT must analyse the realm, individual process and situation. The three realm (individual, organisational and societal) are independent and is possible found relevant information in each one. In this case the problem is within the relationship between the patient, the PT and the family. Although this individual realm is considered the most important, it’s possible to find some societal beliefs about aging. After consideration of the realm is necessary evaluating the moral process involved in the PT himself. In the individual process the PT need a moral judgement to decide what to do about the situation (talk with the client or not), moral motivation (put the ethical value above the family wishes) and moral courage to implement the decision. The final analysis of step one is classify the ethical situation. This case is defined as a dilemma (“Tell the client” or “Respect the family wishes”). Step two allows the reflection of the case, considering the facts (Life and death situation; Family wants to keep the secret), stakeholders opinions, all know about the situation, ethics and professional deontology and the consequences of the decision. In step three of RIPS mode is specifically for the resolution of dilemmas or decide the right thing to do. The PT decision was to inform the patient's diagnosis after talking with the family. In step four exist for define the way to implement the decision The PT consider that is better that this information comes from a physician that could answer other question that the client have about his condition and life perspective.

**Discussion and conclusions:**

Physiotherapist are confronted with a range of ethical and regulatory issues in all practice environments. Decided what is best for the patient is often influenced and, at times, compromised by external factors, like organisations and patient’s family. Physiotherapy as is core of values and ethical principles, but most feel difficulties to identify and analyse ethical situations that happen in his job [2]. The RIPS model, widely spread by APTA, and used as a learning tool for PT students [8] can be useful in large range of situations and contexts. Although there are few publications on the use of this model by physiotherapist. The physiotherapist must be aware of the incapacity process that could result from ageing, on the other hand, notice that this does not necessarily lead to the loss of elderly person autonomy. Respect for the autonomy of the elderly is highlighted as a cornerstone of care by health professionals and family members [6]. Sometimes the risk of paternalism or taking a ‘benevolent decision-making’ in another person’s best interests, is very present, and not so easy to see or discuss with family. This way, the ethical principles of the elderly population are not different from other populations, sharing common rights and duties. The RIPS Model supports an ethical reflection and help to solve ethical issues that physiotherapist could face with the elderly population as showed. The RIPS model has already been used with other populations [7] and demonstrates that it is not the population that defines the ethical approach. The RIPS model supports a strong physiotherapy ethical decision, regardless of the population and clinical context of practice, leading to professional growth.
